# The verb and noun test for peri-operative testing (VAN-POP): standardized language tests for navigated transcranial magnetic stimulation and direct electrical stimulation

**DOI:** 10.1007/s00701-019-04159-x

**Published:** 2019-12-10

**Authors:** Ann-Katrin Ohlerth, Antonio Valentin, Francesco Vergani, Keyoumars Ashkan, Roelien Bastiaanse

**Affiliations:** 1 Center for Language and Cognition (CLCG), University of Groningen, Oude Kijk in ’t Jatstraat 26, 9712 EK Groningen, The Netherlands; 2 International Doctorate for Experimental Approaches to Language and Brain (IDEALAB), University of Groningen, Groningen, The Netherlands; 3 Department of Basic and Clinical Neuroscience, King’s College London, Institute of Psychiatry, Psychology and Neuroscience, London, UK; 4 Department of Neurosurgery, King’s College Hospital, London, UK; 5 Center for Language and Brain, National Research University Higher School of Economics, Moscow, Russia

**Keywords:** nTMS, DES, Language mapping, Object naming, Action naming, Standardization

## Abstract

**Background:**

Protocols for intraoperative language mapping with direct electrical stimulation (DES) often include various language tasks triggering both nouns and verbs in sentences. Such protocols are not readily available for navigated transcranial magnetic stimulation (nTMS), where only single word object naming is generally used. Here, we present the development, norming, and standardization of the verb and noun test for peri-operative testing (VAN-POP) that measures language skills more extensively.

**Methods:**

The VAN-POP tests noun and verb retrieval in sentence context. Items are marked and balanced for several linguistic factors known to influence word retrieval. The VAN-POP was administered in English, German, and Dutch under conditions that are used for nTMS and DES paradigms. For each language, 30 speakers were tested.

**Results:**

At least 50 items per task per language were named fluently and reached a high naming agreement.

**Conclusion:**

The protocol proved to be suitable for pre- and intraoperative language mapping with nTMS and DES.

## Introduction

When treating tumors in eloquent areas of the brain, function-based resection has been proven the best approach to preserve quality of life, while maximizing the extent of resection [12, 20, 35]. Direct electrical stimulation (DES) mapping is traditionally used to identify functional boundaries at cortical and subcortical level and to guide the resection. DES can disturb the neural activity of a small cortical area. If the patient is unable to perform a specific task during stimulation, it is concluded that the stimulated area is involved in a specific cognitive or motor function and should be spared during resection [8, 15]. This procedure is known as the gold standard in decision making for the extent of tumor resection and is currently superior to other methods [12, 13]. During the last decade, DES has mainly been used to locate language functions in patients with a tumor in the left peri-sylvian area; although nowadays it is recommended to monitor other cognitive functions as well, both in the left and the right hemisphere [43]. Another recent development is perceptive language testing for intracranial stimulation in epilepsy patients, for which specific test batteries have been developed [1].

### Navigated transcranial magnetic stimulation

Lately, progress in the functional mapping of language has been made with navigated transcranial magnetic stimulation (nTMS). Using a high-precision coil, matched with neuro-navigation and analytic software, nTMS delivers pulses of magnetic stimulation directed through the skull onto the individual’s cortex. This approach uses the same underlying assumption as DES of revealing functional areas by disrupting neural cortical activity, however, without requiring a craniotomy. Therefore, nTMS mapping delivers valuable preoperative information about cortical language representation to the clinical team to plan the intraoperative procedure. TMS cannot map the subcortical white matter fiber tracts that need to be identified with intraoperative DES. However, the combined use of nTMS and tractography has been recently described to obtain a preoperative mapping of the language network [28, 36, 38]. Having a preoperative map of the functional boundaries depicted by nTMS then leads to a shorter intraoperative procedure [31], a more targeted craniotomy [37] and more confidence in decision making for the surgical team. Moreover, for patients who are not eligible for awake surgery, nTMS is a more precise method for language mapping than fMRI or MEG [19, 40].

### Language testing under stimulation: object naming

Both methods for language mapping, DES and nTMS, require specific tasks that meet the stimulation criteria. The task should trigger a short answer that can be targeted by the stimulation and, hence, show an immediate effect on disturbing the language output. In addition, the task should be sufficiently complex to mimic natural language and challenging enough to capture the individual’s language skills. A task meeting these criteria that is extensively used in many languages is object naming: a picture of an object is shown to the participant for 700–1000 ms, aligned with the stimulation targeting the cortex [23]. The patient is asked to name the picture, sometimes with the lead-in phrase “this is …”. At the cognitive level, this requires retrieval of the noun and its accompanying article and integration of this noun phrase into the sentence by inflecting it for the correct gender, number, and case, in languages where this is applicable. When a language area is stimulated during object naming, the patient may have difficulties carrying out this task and display various kinds of errors, such as anomias, semantic or phonological paraphasias, or full speech arrests. Object naming is easy to administer and straightforward to evaluate. However, a task solely triggering nouns hardly represents natural language output and cannot fully test the individual’s ability to use grammar. Not nouns, but verbs function as the core of a sentence. A task triggering verbs is therefore recommended [32].

Experimental data from several fields of research have confirmed this claim. Firstly, from stroke research, it is known that a task involving nouns is not sufficient to capture linguistic abilities and discover impairments. Cases have been reported of individuals with relatively good production of nouns, but an impairment in tasks involving verbs and verb inflection [6, 22, 26, 27] as well as the inverse, although the inverse is rarer [44]. These observations support the inclusion of both tasks, since the impairment in one of these word classes may go unnoticed if only one task is being used. Secondly, several language-localization studies have shown that cortical areas that do not fully overlap are responsible for carrying out the tasks, object naming and action naming. Even though a shared peri-sylvian network seems to support both tasks, not one task alone can cover all revealed areas ([10, 27, 44], see [32, 42] for a review). Whereas DES to superior frontal, parietal, and temporal regions was reported to elicit errors in both tasks [3, 25], especially the middle and inferior frontal cortical regions as well as subcortical pathways of the long association white matter demonstrated a selective response to verb tasks [2, 3, 25].

Moreover, for some individuals, no positive language areas at all were found under DES mapping using a noun task, whereas a verb test was much more informative [34]. Hence, the need to include a verb task when administering nTMS and DES language mapping became evident.

### Language testing under stimulation: action naming in sentence context

The ideal variant of a verb task is *action naming in sentence context*: the patient is shown a picture of a person or animal carrying out an action and is asked to describe it using a lead-in phrase such as “the man…”, aligned with the stimulation. To produce the correct phrases, the patient has to retrieve the correct verb and integrate the verb into the sentence by inflecting it for the correct person (1st, 2nd, 3rd), number (singular, plural), and tense (past, present, future). This retrieval captures the patient’s application of grammatical rules. Since the task requires many linguistics processes simultaneously, more error types can appear under stimulation for verbs than for nouns: grammatical errors of failed inflection of person, number, or tense can appear. Action naming in sentence context, therefore, is a straightforward tool and as easy to administer as object naming [14, 33]. Moreover, by including grammatical factors, it allows testing the patient’s language skills more thoroughly than an object naming task alone.

These lines of reasoning have led to the development of various tests for intraoperative testing with DES. The Dutch Linguistic Intraoperative Protocol (DuLIP; [14]), for example, offers a wide range of tasks including both noun and verb retrieval which can be selected based on the patient’s characteristics. Whereas some intraoperative batteries such as the aforementioned DuLIP entail action naming tasks, these paradigms were designed to fit the time window of DES, lasting for up to 4 s [13]. One cannot expect the same stimuli and set-up to be suitable for the much shorter stimulation of TMS that is most effective for language mapping with a stimulation of 1–2 s [40]. Similarly, well-established tests such as the Comprehensive Aphasia Test [39], that one may want to employ, were constructed to suit only non-timed testing scenarios. These allow both long answers, consist of few items per task and therefore do not qualify for nTMS or DES mapping either. As yet, no battery fitting the exact parameters of nTMS is available.

### The current study

So far, two studies have looked into verb tasks in nTMS language mapping [17, 18]. Both concluded that the verb task did not reveal more information than the noun task alone. However, both studies employed a simple action naming task that did not involve any sentence context and, thus, no verb inflection. Consequently, such a task does not trigger the grammatical processes mentioned above that constitute the real contribution of a verb task. Moreover, the previous studies used home-made items for the action naming task that had not been standardized, compared to an extensively pretested object naming task. A paradigm consisting of both object naming and the necessary addition of action naming in sentence context with standardized, pretested items is, therefore, needed.

This paper presents the processes of developing, norming, and standardizing the *verb and noun test for peri-operative testing*, the *VAN-POP*. We describe a two-task paradigm for English, German, and Dutch, to be used under both nTMS and DES in a population of different age and backgrounds. For this purpose, an object naming test was designed, as well as two variants of an action naming test, both balanced and controlled for various linguistic factors.

To ensure that the pictures and items elicit the target answer, thorough pilot testing and standardization of the protocol are the first steps necessary to implementing both tests into a protocol for preoperative mapping with nTMS and intraoperative mapping with DES. This paper can function as a guideline for developing similar tests in other languages. The picture stimuli are available for centers that are interested, and assistance is offered by the authors for standardizing the tests in other languages.

## Methods and materials

The VAN protocol consists of two tests: *object naming in sentence context (ON)* and *action naming in sentence context (AN*).

### Materials

#### Object naming

**Fig. 1:**
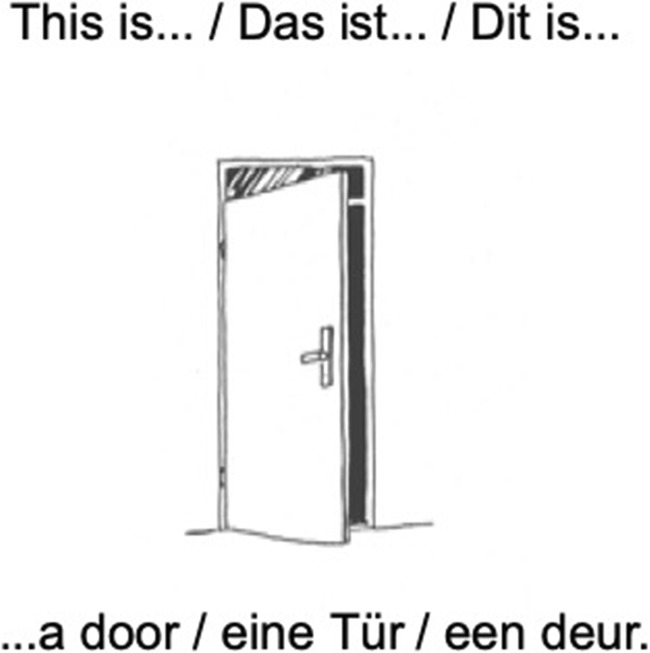
An example of a stimulus for object naming in English, German, and Dutch, respectively. (Artwork by Victor Xandri Antolin; © University of Groningen)

The picture stimuli in the object naming paradigm show black-and-white drawings of everyday objects and animals. Above each picture a lead-in phrase is displayed that introduces the target noun phrase. See Fig. 1 for an example.

In order to provide the correct answer, the participant has to recognize the picture, read the lead-in phrase, retrieve the correct noun and the matching indefinite article, and integrate the noun phrase into the structure of the sentence by satisfying the syntactic requirements (see examples 1–3).

- *English*

This is …a door.

article + noun (number = singular, case = nominative).

- *German*

Das ist ...ein**e** Tür.

article (gender = **female)** + noun (number = singular, case = nominative)

- *Dutch*

Dit is… **een** deur.

article + noun (number = singular, case = nominative)

In English, case is not visible on noun or articles, but the phonological requirement of choosing “a/an” depending on the onset of the following noun has to be met: compare “a car”/“an apple”. For Dutch, there is no overt marking on the indefinite article and the noun. In German, the noun can be masculine, feminine, or neuter and this is marked on the article. Case is always nominative.

#### Action naming

**Fig. 2:**
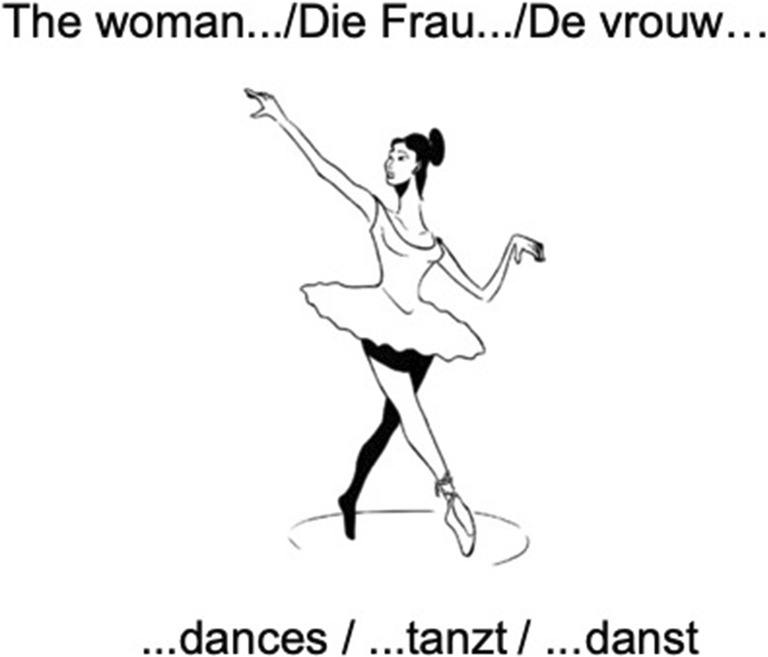
An example of a stimulus for action naming in English, German, and Dutch, respectively. (Artwork by Victor Xandri Antolin © University of Groningen)

The stimuli in the action naming paradigm show black-and-white drawings of a person or animal performing an everyday action, drawn by the same artist as the nouns of the object naming task. Parallel to object naming, every picture displays a lead-in phrase triggering the target verb in sentence context. See Fig. 2 for an example of a picture stimulus with the target sentence in each of the three languages.

To correctly complete the sentence, the participant has to master the following processes: recognizing the picture, reading the lead-in phrase, retrieving the correct verb, and integrating this verb, correctly inflected for person, number, and tense, into the sentence (see examples 4–6).

- English

Daily, she…dance**s**

verb (tense = present, person = **3rd person singular**)

- German

Die Frau…tanz**t**

verb (tense = present, person = **3rd person singular**)

- Dutch

De vrouw…dans**t**.

verb (tense = present, person = **3rd person singular**)

In German and Dutch, these stimuli elicit present tense verbs in a straightforward manner. However, for English, the elicitation of action verbs inflected for person, number, and tense is more difficult to trigger than in German and Dutch. A picture as in Fig. 2 is likely to elicit the present continuous of the verb (“the woman is dancing”). As a result, the verb “to be” would appear with a present participle in each answer. Hence, the inflectional processes targeted vanish, as the intended verb is not inflected for person, number, and tense. Adding the temporal adverb “daily” to the lead-in sentence solves this issue because it elicits the habitual present tense (“dance*s”* in the example). This addition allows an investigation of whether this process is correctly and overtly done even in a morphologically poor language like English. In order to keep the sentences equally long in all languages, personal pronouns were used in English (“he” and “she”) instead of full noun phrases (“the man”/“the woman”).

Moreover, English does not have complex verb inflection. Therefore, a variant of action naming in sentence context was developed: with the lead-in phrase “yesterday, he….” a verb in past tense is triggered. Using this paradigm of action naming past, a more complex process of inflecting the verb is triggered, especially when irregular verbs were included (examples 7 and 8).

- Yesterday he …cook**-ed**.

verb (tense = past, person = 3rd person singular)

- Yesterday he…**ran**.

verb (tense = past, person = 3rd person singular)

### Balancing and sorting

For all target nouns and verbs, relevant values were acquired to allow balancing, controlling, and sorting of the lists of stimuli. For all nouns, the word frequencies were based on the SUBLEX corpora (for English: [41]; for German: [9]; for Dutch: [21]); age of acquisition was based on the Kuperman corpus [24] or if not available, on online questionnaires filled out by native speakers; measures for length in syllables [30] and livingness of the object (animacy) were discussed between the authors based on linguistic theories; the same was done for verbs which were additionally controlled for regularity of the past variant, transitivity, argument structure,^1^ instrumentality (is a man-made instrument needed to perform the action, as, e.g., in "cutting"), and name relatedness to a noun (as, e.g., in a "saw"–"sawing"). Between tests, object and action naming were balanced for word frequency and age of acquisition. The verbs in action naming present and past were balanced for irregularity of verbs in the past tense.

These linguistic factors are known to influence naming accuracy in language-impaired populations (see [7, 42] for an overview and their origin). The characteristics such as age of acquisition (AoA) affect lexical access: the more frequent a word and the earlier it is learned, the easier it is to retrieve. This effect becomes particularly relevant in clinical populations such as tumor patients who may have problems accessing low frequency words. Items with these characteristics can then be removed to tailor the list to the patient’s skills.

Nouns and verbs were matched for word frequency and age of acquisition across lists. For English, verbs with regular and irregular past tense were balanced with respect to all relevant variables. When these balanced lists had been created, the tests were standardized in a two-step procedure, the *pilot phase* and the *standardization phase*.

### Pilot phase

#### Participants

To exclude pictures that were not clear depictions of the target noun or verb, a set of about 100 pictures of objects and about 100 pictures of actions was presented to 5–10 native speakers of each of the three languages. This screening allowed exclusion of unsuitable pictures without testing a large group. The demographics of these groups are given in Table 1.

**Table 1:** Demographics of participants in each language in the pilot phase

	Gender	Mean age	Age range
English	3 female/2 male	29.3	22–48
German	5 female/5 male	34.4	23–61
Dutch	5 female/5 male	34.1	23–63

#### Procedure and scoring

From a database of about 400 available black-and-white drawings, around 100 pictures of nouns and 100 pictures of verbs had been selected for each of the three languages to elicit possible one-word answers in these languages. They were programmed in a Power Point presentation and presented via a laptop. The answers of the participants were audio-recorded.

The laptop was placed in front of the participant displaying the picture stimuli. Participants were instructed to name the picture with the first label that came to mind. At this point of the testing, no time constraint was given for answering. During the session, the responses were scored on a score sheet and later compared to the audio recordings as a check.

#### Results

Only items that were consistently named with one or two labels by at least 80% of the participants were selected for the item lists. Items that elicited too many synonyms were excluded as well (see Table 2).

**Table 2:** Number of items consistently and correctly named by at least 80% of the participants (on the total number of items) per language and per task in the pilot phase

	Object naming	Action naming
English	80/96	74/90
German	80/110	80/100
Dutch	107/130	125/150

A new test was developed with these items, again completely balanced for relevant variables such as word frequency and age of acquisition. For each item, one or two target labels were specified as correct answers, defined by the answers of the participants. In English, the list consisted of 154 items (80 nouns and 74 verbs), in German 160 items (80 nouns and 80 nouns), and in Dutch 160 items (80 nouns and 80 nouns).

### Standardization phase

#### Participants

During the final standardization, the full paradigm was tested on 31 German, 21 Dutch, and 28 English native speakers. The participants were balanced in gender and covered each of five age groups (18–25, 26–35, 36–45, 46–55, 56–65) to ensure that the test items were suitable for every age group of given patients. Since performance at ceiling is expected for the tasks, the group sizes could remain small. Table 3 depicts the demographics of the groups.

**Table 3:** Demographics of participants per language in the standardization phase

	Gender	Mean age	Age range
English	15 female/13 male	40.9	23–65
German	18 female/13 male	43.7	18–75
Dutch	12 female/9 male	39.7	20–73

#### Materials

The roughly 150–200 items that passed the testing phase were included in the standardization phase.

#### Procedure and scoring

Again, the stimuli were inserted in a Power Point presentation and presented on a laptop. The answers of the participants were audio-recorded. The procedure was designed in a way to mimic the situation under DES and nTMS, which means the picture presentation time was 700 ms for the objects and 1000 ms for the actions, as it has been reported that action naming takes more time than object naming. The inter-stimulus interval was set to 2500 ms. Thus, there was a time limit for responding to the stimulus before the next item appeared after 2500 ms.

The participants were sitting comfortably, looking at the screen. They were asked to read the lead-in phrase aloud and complete it using the name of the object or action in the picture as quickly as possible. Also, they were instructed to keep the sentence short by completing it with only one word, the noun or the verb. Each task started with two practice items and if the participant made a mistake here, s/he was corrected. The object naming test was always administered first.

Scoring was the same as in the first phase: the answers of the participants were scored on a score sheet and recorded to check the results later. Items were considered correct if one of the target words was given on time. For some items, it seemed counter-intuitive to the participant to leave out the argument of the verb (“he counts *cash*”), even though instructed to do so. These cases were registered, but the items were not excluded.

### Analysis

For each item, it was noted, whether at least 80% of the participants had answered with one of the target words within the given time. In that case, the item was considered to have passed the *naming agreement threshold*.

For testing differences in excluded items per test that did not pass the threshold, Barnard’s tests were performed. Naming accuracy of all items after exclusion was established by calculating the correct answers per task and compared by using Mann-Whitney *U* comparisons.

Differences in naming accuracy by age group were compared by applying Kruskal-Wallis rank sum tests to the defined age groups (18–25, 26–35, 36–45, 46–55, and 56–65). Furthermore, to evaluate correlations between the linguistic factors and naming accuracy, Spearman correlations with a two-tailed *p* value and 95% confidence interval were performed. Bonferroni corrections were applied to account for multiple comparisons for correlations.

## Results

After excluding all items that did not pass the naming agreement threshold of 80%, the final lists resulted in about 50 items for both the object naming and the action naming test in each language. Table 4 summarizes the exact numbers of resulting items per language and test.

**Table 4:** Number of items included (on the number of items tested) in the final version of the VAN-POP after the standardization phase

	Object naming	Action naming	Present	Past
English	50/80	50/74	23/37	27/37
German	75/80	75/80	–	–
Dutch	75/80	75/80	–	–

Some further items in the object naming task were excluded to equalize the final item numbers across tasks. There was a higher number of excluded items for the action naming task compared to the object naming task in all three languages (English: *p* < 0.01, German: *p* = 0.02, Dutch: *p* = 0.02). No difference was found for items excluded in the present and past tense variant for action naming in the English test (*p* = 0.46).

A significantly higher naming accuracy was found for object naming than for action naming in all three languages (*p* < 0.01), see Table 5, as well as a significantly higher naming accuracy in action naming present compared to action naming past (*p* = 0.013).

**Table 5:** Naming accuracies for the subtasks of the VAN-POP after exclusion

	Object naming	Action naming	Action naming present	Action naming past	*p* value
English	0.989	0.91			*p* < 0.01
			0.947	0.878	*p* = 0.013
German	0.952	0.90			*p* < 0.01
Dutch	0.975	0.923			*p* < 0.01

The correlations between the linguistic factors and the naming accuracies in each of the tests are summarized in Appendix A, Table 7. Correlations were solely found in Dutch: AoA correlated negatively with naming accuracy in object naming (*r* = − 0.2996, *p* = 0.036) and action naming (*r* = − 0.3357, *p* = 0.0256).

None of age groups performed significantly better or worse than the other in any of the languages, as summarized in Table 6.

**Table 6:** Kruskal-Wallis rank sum tests for differences in naming accuracies between age groups per language

	*X*^2^	*p*
English		
Object naming	3.34	0.34
Action naming present	0.76	0.94
Action naming past	9.89	0.27
German		
Object naming	10.767	0.22
Action naming	18.214	0.08
Dutch		
Object naming	8.823	0.12
Action naming	12.268	0.43

## Discussion

This study was developed to design a task paradigm for pre- and intraoperative language testing to examine the patient’s skills more comprehensively than the standard one-task paradigm using object naming. We proposed, that next to object naming, action naming in sentence context is not only useful (as already shown by [34]), but also a necessary addition to language mapping protocols. Building on this assumption, we present a test paradigm of a balanced combination of object and action naming in three languages that can more accurately test and potentially map language.

### Object versus action naming

The proposed test, the VAN-POP, was validated in a group of around 30 native speakers per language that covered a variety of age groups and backgrounds. In each of the languages, after exclusion of items with a naming agreement of less than 80%, each of the tests resulted in a final item list of at least 50 items. Thus, the tasks have proven to consist of reliable items, that the participants reacted to correctly and in the time given, under presentation parameters mimicking those with nTMS or DES mapping. Age did not influence performance, indicating that the task is simple and can be reliably administered in any native speaker of the three languages.

When looking into the accuracy rates after exclusion of poor items, differences between tasks did surface, that give insight into future application of the VAN-POP under stimulation. Action naming delivered an overall lower accuracy rate than object naming. This finding may be due to the higher visual complexity of the pictures that comes with the nature of an action depicted by a verb. Object naming can be elicited with depicting the object to be named in isolation, whereas the pictures of actions show at least the person carrying out the action, often accompanied with an object necessary to carry out that action or a background needed to identify the action. Given the short time frame of presentation, 1000 ms, visual complexity may have led to more errors on the action naming test. Secondly, action naming yielded more variable answers than object naming, resulting in lower accuracy scores. This finding may be caused by the tendency of some participants to paraphrase the answer with a light verb ("he climbs" -> "he does climbing"); or by using a synonym of the verb that does not capture the targeted action ("he sweeps" -> "he cleans"). While those examples are adequate descriptions of the picture, they are harder to score, when later comparing them to answers under stimulation and were, therefore, counted as an error in the analysis.

The factors named above deliver valuable feedback about administering the tasks. Even with clear instructions, more variation must be expected in the answers to the action pictures. Both less precise choices of verbs and paraphrasing could arise, and a decision has to be made per individual, whether or not to count the response as an error. Paraphrasing the target may be a sign of avoiding verb inflection or a strategy to hide word-finding problems, especially in a clinical group. Using a light verb construction may hint towards already existing impairments to grammatical skills and should be noted. Consequently, action naming is a more complex task, both visually and linguistically, reflected in a lower accuracy, even though the items on both tests were carefully balanced with respect to relevant factors such as word frequency and age of acquisition. However, this complexity does not decrease the quality of the single items in the tests. It underlines individual differences among the participants. The action naming test remains a robust tool in addition to the object naming, with a high naming agreement.

### Action naming variants

Keeping in mind that the contribution of action naming is to test grammatical skills seen in inflection on the verb, we argue that it is useful to add a variant of action naming that requires more inflectional effort in English. Action naming past is, thus, part of the English protocol.

Nonetheless, similar to the accuracy rates of object versus action naming, the accuracy for action naming past was lower than for the present variant. The reason for this may be the higher cognitive load for inflection for past, as earlier shown in agrammatic aphasia [5]. Again, the higher linguistic complexity being required for inflecting the verb for past versus present becomes evident in the accuracy rate, but it does not influence the validity of the test. Rather, action naming past offers a second, more complex version of a verb task to tap into grammatical skills.

### Linguistic variables of naming

The majority of linguistic variables such as word frequency and animacy of the object did not show a correlation to naming accuracy. Solely a low age of acquisition was negatively correlated with a higher naming accuracy in Dutch for both object and action naming. These findings are in line with the aphasiological literature showing that factors such as the age at which a word is learned in childhood (low AoA: [7, 11, 29]) play a role in access and retrieval of verbs and nouns, and more importantly, can be affected differently due to brain damage, also when caused by a tumor. With the VAN-POP, it is possible to identify which factors influence noun and verb retrieval and a proper patient-tailored version of the test can be made that is still balanced for relevant factors.

Compared to former studies with nTMS and DES employing different tasks [17, 18], two factors set our study apart from the existing ones: firstly, our study used thorough balancing, scoring, and pilot testing of the items and lists to ensure a higher quality of the new tasks. The second factor is the explicit inclusion of a lead-in phrase in writing above each picture and the triggered target in sentence context to test grammatical skills. Our study is the first to validate this approach for presentation parameters of nTMS and DES with balanced item lists.

### Future directions

The VAN-POP test has been designed to be used in clinical practice. Further studies are required to test its validity under nTMS in healthy participants and under nTMS and DES in neurosurgical patients. The stimuli qualify for both settings, as the current study showed that the pictures can be named in sentence context within a time frame of 1000 ms, the usual presentation time under nTMS. The stimuli are suitable for DES as well, where usually both the stimulation duration and stimulation presentation times are 4000 ms [4, 13, 16]. In both stimulation settings, the tasks in sentence context can then be used to reveal language areas through error elicitation. Furthermore, we do not disregard the usage of the VAN-POP for other clinical interventions, such as epilepsy surgery. Future studies will have to examine the paradigm further by using it under stimulation. To do so and to make the VAN universally applicable, all picture items are made available to interested centers. Adaption to other languages than the three described here is highly encouraged by the authors, who offer their assistance in doing so.

## Conclusion

The VAN-POP protocol, available in English, German, and Dutch, delivers a picture naming paradigm to test retrieval and production of both nouns and verbs in sentence context. The protocol proved to be suitable for peri-operative mapping parameters, such as those for nTMS and DES language mapping. In each language, at least 50 times per test passed the standardization phases and are ready to be used.

## Appendix

**Table 7:** Correlations (Spearman rank) between linguistic factors and naming accuracies in tests per language

	rho	*p* value after Bonferroni corrections
English		
Object naming		
Frequency	0.1285691	0.9999
AoA	− 0.2480907	0.3292
Length	0.1947115	0.7016
Animacy	0.08952827	0.2256
Action naming present		
Frequency	0.2797524	0.9999
AoA	− 0.3125268	0.9999
Length	0.2336326	0.9999
Transitivity	− 0.3322092	0.9712
Number of arguments	− 0.3322080	0.9712
Instrumentality	− 0.271525	0.9999
Name relatedness to noun	− 0.1324906	0.9999
Action naming past		
Frequency	0.09020308	0.9999
AoA	− 0.3821577	0.3936
Length	− 0.3447656	0.6256
Regularity	− 0.04077954	0.9999
Transitivity	0.0067992	0.9999
Number of arguments	0.006798	0.9999
Instrumentality	0.03877786	0.9999
Name relatedness to noun	0.3070675	0.9536
German		
Object naming		
Frequency	0.04850672	0.9999
AoA	− 0.2034235	0.3200
Length	0.0461811	0.9999
Animacy	0.138077	0.9500
Action naming		
Frequency	0.1540224	0.9999
AoA	− 0.2893704	0.0944
Length	− 0.01664306	0.9999
Transitivity	− 0.02537652	0.9999
Number of arguments	− 0.04504365	0.9999
Instrumentality	− 0.07385802	0.9999
Name relatedness to noun	− 0.01873735	0.9999
Dutch		
Object naming		
Frequency	0.09742688	0.9999
AoA	− 0.2995647	0.0360
Length	− 0.2112492	0.2756
Animacy	0.03880498	0.9999
Action naming		
Frequency	0.2316214	0.3648
AoA	− 0.3357215	0.0256
Length	− 0.1349784	0.9999
Transitivity	− 0.0249946	0.9999
Number of arguments	− 0.02794969	0.9999
Instrumentality	− 0.1284161	0.9999
Name relatedness to noun	− 0.1481571	0.9999

## Abbreviations

AN: Action naming
AoA: Age of acquisition
DES: Direct electrical stimulation
DuLIP: Dutch Linguistic Intraoperative Protocol
nTMS: Navigated transcranial magnetic stimulation
ON: Object naming
VAN-POP: Verb and noun test for peri-operative testing

## Footnotes

1 All verbs were action verbs; parts of them were intransitive and had one argument, the agent (e.g., *to swim)*; parts of them were pseudo-transitive, meaning they have a theme that does not need to be produced to create a grammatical sentence (e.g., *to read*).
